# Understanding the Role of Sensorimotor Beta Oscillations

**DOI:** 10.3389/fnsys.2021.655886

**Published:** 2021-05-31

**Authors:** Jacopo Barone, Holly E. Rossiter

**Affiliations:** Cardiff University Brain Research Imaging Centre, School of Psychology, Cardiff University, Cardiff, United Kingdom

**Keywords:** beta rebound, beta desynchronization, beta bursts, brain oscillations, sensorimotor processing, functional role

## Abstract

Beta oscillations have been predominantly observed in sensorimotor cortices and basal ganglia structures and they are thought to be involved in somatosensory processing and motor control. Although beta activity is a distinct feature of healthy and pathological sensorimotor processing, the role of this rhythm is still under debate. Here we review recent findings about the role of beta oscillations during experimental manipulations (i.e., drugs and brain stimulation) and their alteration in aging and pathology. We show how beta changes when learning new motor skills and its potential to integrate sensory input with prior contextual knowledge. We conclude by discussing a novel methodological approach analyzing beta oscillations as a series of transient bursting events.

## Introduction

**FIGURE 1:**
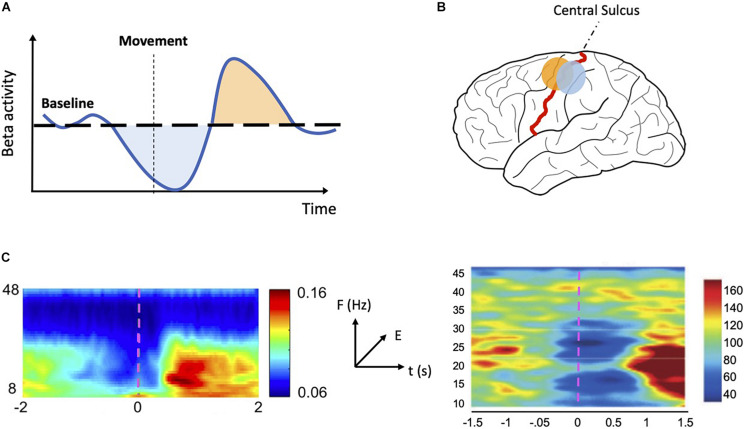
MRBD and PMBR. **(A)** Schematic representation of sensorimotor beta activity. During a motor task beta activity drops below baseline (MRBD—*light blue shaded area*) just prior to and during movement execution. After movement ends, beta activity increases rapidly (PMBR—*light orange shaded area*) before slowly returning to baseline level. **(B)** Schematic representation of MRBD and PMBR spatial distribution. MRBD (*light blue dot*) is commonly localized to the postcentral gyrus whereas PMBR (*light orange dot*) is often localized to the precentral gyrus. **(C)** Time-frequency plots showing MRBD and PMBR in M1 (*left*—reproduced and adapted with permission from Little et al., 2019) and STN (*right*—reproduced and adapted with permission from Alegre et al., 2005). Both plots show oscillatory activity changes specific to the beta band (13–30 Hz) in the period prior to movement and following movement termination. The color scale indicates relative energy changes with respect to baseline level (blue colors indicate a decrease; red colors indicate an increase). The movement begins at time 0 (*magenta dashed line*). MRBD: movement related beta decrease; PMBR, post-movement beta rebound; M1: primary motor cortex; STN, subthalamic nucleus.

Recordings of electrical signatures of the brain, *via* electroencephalography (EEG), magnetoencephalography (MEG), electrocorticography (ECoG), and local field potential (LFP), have consistently reported rhythmic patterns in neural activity. Brain rhythms, also known as oscillations, are linked with numerous cognitive functions and group into several oscillatory bands (Buzsáki, 2006). Beta oscillations (∼13–30 Hz) are commonly implicated in sensorimotor processing (Pfurtscheller and Lopes da Silva, 1999; Baker, 2007). These oscillations are established during stable postures and are decreased during active states, such as movement planning and execution (Engel and Fries, 2010; Kilavik et al., 2013). A decrease in the amplitude of beta oscillations across sensorimotor areas is seen just prior to and during movement execution. Conversely, an increase of beta amplitude above baseline levels is observed following movement cessation. We refer to these two phenomena as movement related beta decrease (MRBD) and post-movement beta rebound (PMBR), respectively (Figure 1A). The two principal sources of beta are sensorimotor cortex (Jensen et al., 2005; Roopun et al., 2006; Kramer et al., 2008; Yamawaki et al., 2008; Kopell et al., 2011) and basal ganglia (Figure 1C; Holgado et al., 2010; McCarthy et al., 2011; Tachibana et al., 2011; Mirzaei et al., 2017). There is debate as to whether they originate independently in each area or if they are an emergent property of the cortico-basal ganglia networks (Pavlides et al., 2015; Sherman et al., 2016; Reis et al., 2019).

The scope of this review is to report the most recent interpretations of the role of beta oscillations in the sensorimotor system. We will do so by giving details of how beta oscillations are affected by healthy aging (Rossiter et al., 2014b) and motor learning (Tan et al., 2014, 2016; Torrecillos et al., 2015; Haar and Faisal, 2020) as well as how they are altered in various conditions, most notably Parkinson’s disease (PD) and stroke, characterized by severe motor symptoms (Brown, 2006; Rossiter et al., 2014a). We will also cover how beta oscillations can be manipulated experimentally using drugs and brain stimulation techniques. We will conclude by discussing how assessing the transient nature of beta oscillations could enrich our understating of its functional role (Feingold et al., 2015; Sherman et al., 2016; Shin et al., 2017).

## Functional Role of Beta Oscillations

Although several studies have extensively investigated beta oscillatory dynamics (Table 1) [for review, see Kilavik et al. (2013)], its functional role in the sensorimotor processing is still not fully understood. One source of interest lies in beta’s tendency to fluctuate during movement. MRBD is present during spontaneous and triggered movements (Kilavik et al., 2013), while successful movement cancelation is associated with an increase in beta (Swann et al., 2009, 2012; Wagner et al., 2018; Jana et al., 2020). MRBD also occurs when no muscle contraction is required (i.e., motor imagery or action observation) and is rather insensitive to parameters like movement type or effector (Miller et al., 2010; Kilavik et al., 2013). These observations led Engel and Fries (2010) to propose a role for beta as an active process, which interferes with the encoding of incoming information while promoting the existing state—i.e., “status-quo”—of the system. Therefore, instead of being a proxy for the level of activity of the sensorimotor network, beta oscillations act as a top-down inhibitory rhythm during motor and cognitive tasks. Following the “status-quo” hypothesis, MRBD and PMBR could be interpreted as endogenous fluctuations of beta level during a motor set, with the former necessary for releasing the inhibition and allowing the initiation of a motor plan, while the latter preserves the existing motor states from internal and external sources of noise.

**TABLE 1:** Selective list of studies describing beta’s functional role in the sensorimotor system.

	**Authors**	**Locus**	**Function**
MRBD	Engel and Fries, 2010	Cortex/Basal Ganglia	Motor plan initiation
	Jana et al., 2020	Cortex	Action stopping
	Leventhal et al., 2012	Basal Ganglia	Gating
	Little and Brown, 2014	Basal Ganglia	Motor impairments
	Shin et al., 2017	Cortex	Attention, perception
	Stolk et al., 2019	Cortex	Inhibition/excitation
	Torrecillos et al., 2018	Basal Ganglia	Motor control
PMBR	Baker, 2007	Cortex/Basal Ganglia	Movement outcome
	Cassim et al., 2001	Cortex	Somatosensory processing
	Engel and Fries, 2010	Cortex/Basal Ganglia	Motor states protection
	Feingold et al., 2015	Basal Ganglia	Task performance, reward
	Fry et al., 2016	Cortex	Inhibition, motor control
	Haar and Faisal, 2020	Cortex	Motor learning
	Tan et al., 2014, 2016	Cortex	Predictive coding
	Torrecillos et al., 2015	Cortex	Error detection

*The studies are split based on whether they focus more on MRBD (*upper half*) or PMBR (*bottom half*). MRBD: movement related beta decrease; PMBR: post-movement beta rebound.*

Post-movement beta rebound has also been interpreted more specifically as an indicator of movement outcome processing (Baker, 2007). Supporting evidence stems from findings showing PMBR is modulated by passive movements (Cassim et al., 2001; Alegre et al., 2002) and by kinematic errors (Tan et al., 2014). More recently, Tan et al. (2016) reported that the level of PMBR over the sensorimotor cortex serves as an index of confidence in the prediction of a motor outcome, also known as the forward model.

Together, these studies propose a role for sensorimotor beta oscillations encompassing multiple functions. We suggest several reasons why interpretations of beta’s role are so varied.

First, beta involves several types of oscillations in distinct frequency bands (Kopell et al., 2011). PD studies show that oscillatory activity through the cortico-basal ganglia network is segregated into low (14–20 Hz) and high beta frequencies (>24 Hz) both in humans (López-Azcárate et al., 2010; Litvak et al., 2011) and rats (West et al., 2018). Furthermore, dopamine levels affect low and high beta rhythms differently (Brown et al., 2001; Priori et al., 2004; Marceglia et al., 2006). One theory proposes that low-beta has an “anti-kinetic” role (Brown, 2003; Chandrasekaran et al., 2019), while high-beta reflects attention and sensory cue anticipation (Saleh et al., 2010; Kilavik et al., 2014; Chandrasekaran et al., 2019).

Secondly, some studies have observed that MRBD and PMBR have a different spatial distribution (with MRBD localizing to postcentral gyrus and PMBR to precentral gyrus) and could represent independent events (Figure 1B; Jurkiewicz et al., 2006; Miller et al., 2007; Alegre et al., 2008; Gaetz et al., 2011; Muthukumaraswamy et al., 2013).

Finally, recent studies suggest that beta shows a burst-like activity, rather than being sustained over time. Therefore, theories of its functional role should integrate this transient nature and the potential implications for behavior (see section “Beta Oscillations as a Transient Rhythm”).

## Natural Variation of Beta Oscillations

### Motor Learning

A group of recent studies (Tan et al., 2014, 2016; Torrecillos et al., 2015; Alayrangues et al., 2019; Haar and Faisal, 2020) strengthen the link between beta oscillations and sensorimotor processes by testing for the role of beta in the context of motor learning. Motor learning can be defined as an improvement of motor skills through practice which is paralleled by long-lasting changes at the level of neural circuitry (Sanes and Donoghue, 2000; Muellbacher et al., 2002; Halsband and Lange, 2006). Motor learning paradigms involve goal-directed actions toward a target (reaching, pointing), while motor performance and/or sensory feedback (force fields, prisms) are experimentally manipulated [for review, see Shadmehr et al. (2010)].

In a visuomotor rotation task, Tan et al. (2014, 2016) found that larger PMBR amplitude indicates more confidence in the forward model and the maintenance of more stable motor output, while smaller PMBR indicates the need for adaptive changes driven by sensory feedback. Recently, Haar and Faisal (2020) explored PMBR dynamics during a real-world billiards task. Across the experiment, PMBR amplitude exhibited opposite modulations, with some participants showing a reduction with learning while others showed an increase. The authors speculated that participants may opt for distinct learning strategies to complete the task during real-world paradigms. Therefore, opposing PMBR dynamics could be interpreted as neural signatures of separate underlying learning mechanisms.

The link between PMBR and motor learning was also explored by Torrecillos et al. (2015). In a pointing task, the authors contrasted two types of reach errors: movement-execution errors that triggered adaptive mechanisms and errors that elicited no sensorimotor adaptation. PMBR amplitude was reduced after experiencing both kinds of errors, leading the authors to suggest a non-specific role for PMBR in error/mismatch detection. In a subsequent study, Alayrangues et al. (2019) contrasted bimanual reaching tasks with comparable motor kinematics but different action goals. Although each task required distinct sensorimotor remapping following a mechanical perturbation, PMBR modulation was comparable across tasks. This finding supports the notion that PMBR is related to salient error-detection mechanisms which act without triggering adaptive behavioral adjustments.

While beta’s role in motor learning is still unresolved, new evidence suggest a complex relation of PMBR with outcome processing. Future studies need to clarify if the observed PMBR modulations during learning could be linked with changes in the upcoming motor output (in agreement with the “status-quo” hypothesis) or may reflect high-level sensory integration processes.

### Aging

Motor performance is generally found to decline with age alongside concurrent changes in beta. Resting beta power has been shown to increase in older adults (Rossiter et al., 2014b; Heinrichs-Graham and Wilson, 2016; Espenhahn et al., 2019) as well as in youth (9–14 years) compared to adults (Heinrichs-Graham and Wilson, 2016). An enhanced MRBD has been seen with increasing age both in older adults compared to younger adults (Heinrichs-Graham and Wilson, 2016; Provencher et al., 2016; Espenhahn et al., 2019) and in children compared to adults (Gaetz et al., 2010). MRBD was also found to reduce after motor learning (Gehringer et al., 2019). These studies suggest that concurrent increase in resting beta oscillations and MRBD may make the release of inhibition to initiate movement more difficult with aging.

From youth to adulthood, an increase in PMBR has been demonstrated (Gaetz et al., 2010) potentially linking to adults improved accuracy and ability to predict motor outcomes as found in Tan et al. (2016). There seems to be a link between changes in beta rebound and how people learn with age. Both Mary et al. (2015) and Moisello et al. (2015) found an enhanced PMBR due to learning in the contralateral sensorimotor cortex in younger adults which was not altered in older adults (Mary et al., 2015) or in PD patients (Moisello et al., 2015). Both authors suggest this may reflect plasticity in the younger adults allowing them to manipulate their beta levels during learning which was not observed in the older subjects or patients. Not only are resting beta oscillations stronger in older adults but it is harder for their PMBR to be altered in response to motor learning.

### Parkinson’s Disease and Stroke

Changes in sensorimotor beta oscillations have been observed in pathology such as PD and following stroke. Brown (2006) hypothesized that strong beta oscillations recorded from basal ganglia structures in PD patients may be antikinetic. Little and Brown (2014) did indeed find a strong correlation between beta and bradykinesia in PD patients. Contrastingly, a study by Heinrichs-Graham et al. (2014) demonstrated a reduced amplitude of beta oscillations in primary motor cortex of PD patients. They suggest this could be due to inhibitory drive originating from basal ganglia areas *via* the thalamus. When modulating dopamine levels in PD patients using levodopa, a suppression of beta power was seen in the subthalamic nucleus (Little et al., 2013) whereas an increase in beta power was seen in the motor cortex (Cao et al., 2020). These results fit with the theory that stronger beta amplitude, in this case in the basal ganglia, maintains the “status-quo” at the expense of voluntary movement although it highlights important differences between cortical and subcortical areas of the network.

Another neurotransmitter, GABA, is altered following stroke which affects levels of plasticity in the brain (Carmichael, 2012). Investigating beta following stroke can potentially give insight into these dynamics. Thibaut et al. (2017) found that higher resting beta power in the affected hemisphere of stroke patients was associated with poorer motor function whereas the reverse relationship was found in the unaffected hemisphere. Rossiter et al. (2014a) found a markedly diminished MRBD in stroke patients with motor impairment compared to healthy controls, and within the patient group, there was a correlation between MRBD and impairment, with reduced MRBD indicating greater level of motor impairment. Not only that but in Espenhahn et al. (2020), stroke patients’ beta parameters were found to be modulated less following motor training than healthy controls. It is possible that stroke makes it more difficult for patients to modulate and suppress their beta oscillations in order to perform movements.

## Experimental Manipulation of Beta Oscillations

### Pharmacological Manipulation

In order to assess the function of beta, it is possible to manipulate it experimentally. One such way of achieving this is through drugs. Changes to both resting state and motor task related activity in the beta band have been seen in response to drugs. At rest, giving benzodiazepines that increase the effect of GABA, such as lorazepam/diazepam, demonstrates a large increase in beta amplitude (Fingelkurts et al., 2004; Jensen et al., 2005; Hall et al., 2010). Studies assessing the effect of GABAergic drugs during simple finger movements demonstrated an increased resting beta power and an enhanced MRBD. PMBR was reduced by tiagabine (a non-specific GABA reuptake inhibitor) (Muthukumaraswamy et al., 2013) but was left unaltered by diazepam which is specific to GABA-A receptors (Hall et al., 2011) suggesting a potentially different mechanism behind MRBD and PMBR. Gaetz et al. (2011) demonstrated the link between GABA and beta oscillations using magnetic resonance spectroscopy and showed a positive correlation between levels of GABA in M1 and PMBR amplitude across individuals. These studies strongly suggest that the amplitude of beta oscillations is linked to levels of GABAergic inhibition in the brain.

### Brain Stimulation

Brain stimulation is another method of manipulating beta in order to understand its underlying mechanisms in greater detail. In order to determine a causal role for beta oscillations, some studies have used transcranial alternating current stimulation (tACS) which stimulates superficial areas of cortex at specific frequencies in order to entrain neuronal firing. Pogosyan et al. (2009) were the first to show that beta frequency tACS over sensorimotor cortex slowed movement. This finding was backed up by Joundi et al. (2012) and Wach et al. (2013), with the latter showing that not only did beta tACS reduce the rate of force development, but gamma frequency tACS also increased it. Brain stimulation techniques offer a unique way of manipulating oscillations in the cortex in order to explore causal links with movement, however, positive findings are often hard to replicate (Héroux et al., 2017). Deep brain stimulation is a therapy in which areas of the basal ganglia are stimulated at high frequency. It can alleviate motor symptoms of disorders such as PD whilst simultaneously suppressing alpha and beta band activity across widespread areas including sensorimotor cortex and basal ganglia (Eusebio et al., 2011, 2012; Tinkhauser et al., 2017; Abbasi et al., 2018; Luoma et al., 2018). These studies point to a potential causal role of beta suppression in order to initiate movement.

## Beta Oscillations as a Transient Rhythm

In previous reports, beta oscillations were typically considered as a repeated cycle of oscillatory activity sustained over time. A growing number of studies, however, are reconsidering beta oscillations as brief bursts of temporally localized activity (Feingold et al., 2015; Sherman et al., 2016; Shin et al., 2017; Tinkhauser et al., 2017; Torrecillos et al., 2018; Little et al., 2019). The idea of oscillations sustained across time stems from the standard analysis procedure of averaging oscillatory activity across many repeated trials (Jones, 2016). Sherman et al. (2016) analyzed source-localized human MEG data looking at spontaneous activity during rest in primary somatosensory cortex. In single trials, beta emerged transiently as a sudden increase in power typically lasting < 150 ms, but when the same data was averaged over many trials, continuous oscillations appeared in the time-frequency spectrogram. Feingold et al. (2015) showed how beta-band events in LFPs occurred predominantly in brief bursts both in the motor-premotor cortex and in the striatum of monkeys performing self-timed movement tasks. Single trial analysis revealed that variations in averaged oscillatory power were expressed by variations in burst density of beta events. Thus, the authors suggest, beta synchronization and desynchronization reflect the probability of occurrence of a brief bursting event, rather than representing modulation of the strength of a sustained oscillation. Finally, Shin et al. (2017) showed that the rate of transient pre-stimulus beta events in the primary somatosensory cortex was the most consistent predictor of stimulus detection in humans and mice, while also being strongly correlated with average beta power. Taken together, cumulative evidence across brain areas, recording modalities and species support the notion of beta as a transient event. Trial averaged beta may conceal the functional importance of the underlying bursting activity, offering a less detailed interpretation of beta’s role in the sensorimotor system. Whilst the averaged amplitude of beta power does seem to correlate with rate of beta events, implying that this new analysis may not contradict what has gone before, it will allow for more detailed exploration which may in turn help to consolidate the details of beta’s role in sensorimotor integration.

## Conclusion

Many of the studies in this review link to the idea that beta oscillations at rest ensure stability and the “status quo” of the motor system. Differently, studies focusing on PMBR highlight a much more nuanced relationship with movement, potentially encoding features of motor learning and error-salience. Although beta’s role is still not fully elucidated, it is unlikely to reflect pure sensory or motor processes. Instead, beta has become more broadly implicated in endogenous top-down processing and sensorimotor integration.

Future studies should work toward establishing a causal role of beta in the sensorimotor system. We described two distinct methods, pharmacology and brain stimulation, that can be effectively used to manipulate beta and infer causality. Furthermore, studies with patient groups with selective beta impairments, such as PD and stroke, can provide invaluable evidence of the physiological relevance of beta in motor control and motor learning. What should also be at the forefront in our minds is the richness of detail that may be gleaned with single trial analysis of beta events, which could return additional features to characterize and understand beta’s role in the sensorimotor system.

## Author Contributions

Both authors contributed to the conception and design of the review, to the interpretation of the relevant literature, and to the writing and editing of the manuscript.

## Conflict of Interest

The authors declare that the research was conducted in the absence of any commercial or financial relationships that could be construed as a potential conflict of interest.
